# Measuring catalytic mechanism similarity – a new approach to study enzyme function and evolution

**DOI:** 10.1111/febs.70106

**Published:** 2025-04-22

**Authors:** Antonio J. M. Ribeiro, Ioannis G. Riziotis, Neera Borkakoti, Pedro A. Fernandes, Maria J. Ramos, Janet M. Thornton

**Affiliations:** ^1^ LAQV‐REQUIMTE, Departamento de Química e Bioquímica Faculdade de Ciências da Universidade do Porto Portugal; ^2^ European Bioinformatics Institute Cambridge UK

**Keywords:** enzyme catalysis, enzyme evolution, enzyme mechanism, enzyme reaction, enzyme similarity

## Abstract

Similarity measures for protein sequence, structure and enzyme reactions have been essential tools for translating an abundance of experimental data about enzymes into biological insights. Enzymes with similar sequence and structure, for example, can be organised into evolutionary families, and within families, reaction similarity can highlight examples of conservation or divergent evolution. When it comes to reaction mechanisms, despite their importance in explaining the catalytic power of enzymes and their evolution, no similarity measures have been developed until now. We addressed this gap by developing a method to calculate mechanism similarity based on the bond changes and charge transfers occurring at each catalytic step, where we have the ability to adjust the size of the chemical environment surrounding the atoms directly involved in these transformations. Using this newly developed method, we performed a pairwise comparison of all the mechanisms stored in the Mechanism and Catalytic Site Atlas (M‐CSA) database. This analysis illustrates how mechanism similarity can be a powerful tool to navigate the known catalytic space and to discover and characterise both convergent and divergent evolutionary relationships.

## Graphical abstract

We introduce a previously undescribed computational method to measure the similarity of enzyme mechanisms, enabling comparison of catalytic steps across hundreds of enzymes. Applying this method to a database of enzyme mechanisms demonstrated its ability to automatically uncover functional and evolutionary relationships, independently of sequence and structure. As the number of characterized mechanisms grows, methods like ours will be essential for understanding enzyme catalysis, supporting classification, and guiding engineering applications.

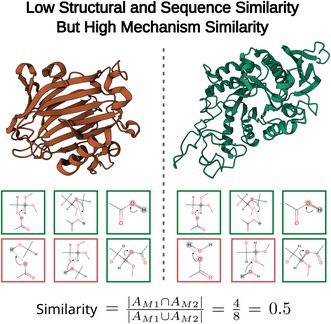

**Abbreviations**
API: application programming interface
EC: enzyme commission number
M‐CSA: mechanism and catalytic site atlas
SMARTS: smiles arbitrary target specification

## Introduction

The volume of biological data pertaining to enzymes, as made available through the literature and specialised databases, keeps growing at an accelerating rate [1]. UniProt [2] currently includes more than 42 million protein sequences annotated with at least one of 6843 EC numbers [3, 4], 12 158 KEGG reactions [5], and 16 970 RHEA reactions [6]. The ability to automatically compare enzymes is essential for understanding this wealth of information, and methods that focus on one of the multiple facets of enzymes—such as their sequence, structure, ligand binding, active site, or chemical reaction—have been extensively used.

Among other applications, similarity measures support the categorisation of enzymes into families. For example, PFAM [7] and CCD [8] use sequence similarity to define families of protein domains that are evolutionarily related, but might have different functions. CATH [9], SCOPe [10] and ECOD [11] use structural similarity to define and categorise structural domains into defined folds. These classifications facilitate the annotation and function identification of experimentally uncharacterised homologous proteins, which comprise the vast majority of UniProt (less than 1% of the proteins in UniProt have been experimentally characterised or manually annotated). A complementary approach for function identification is to focus solely on the active site [12], rather than the global protein structure. At this local level, convergent evolution is also relevant [13] and so methods based on active site similarity can equally work for non‐related enzymes.

Other similarity measures are applicable to the chemical domain of enzymes, including their small molecule substrates and reactions. The comparison of the topological and three‐dimensional representations of ligands is key for the creation of pharmacophores used in drug development [14], or to study the catalytic capabilities of multifunctional enzyme families [15]. Finally, reaction similarity, which might encompass the comparison of the reactants and products of two enzymes or the direct comparison of the bond changes associated with two reactions [16, 17], is crucial for the understanding of enzyme evolution [18] and for the identification of good starting points for campaigns of enzyme engineering [19].

The enzyme mechanism is the sequence of bond changes and other molecular rearrangements that drive the reaction from the substrates to the products. The mechanism provides the central explanation for how the enzyme structure, and particularly the active site, can catalyse the enzymatic reaction. Despite this significance, as far as we are aware, no methods have been developed specifically for comparing enzyme mechanisms so far. This may be attributed to the difficulty in defining mechanisms experimentally, leading to a comparatively lower number of mechanisms described in the literature in comparison to other types of data (such as sequence, structure and reactions) and to the lack of a standardised format for reporting and storing data about enzyme mechanisms.

In order to solve these problems and to support other studies involving enzymes, along the years, we have been collecting examples of enzyme mechanisms and active sites, as reported in the literature, in the M‐CSA (Mechanism and Catalytic Site Atlas) database [20]. Currently, the M‐CSA contains the detailed description of 734 enzyme mechanisms which are stored in a machine‐readable format and are the foundation of the present work. To avoid redundancy, each entry in M‐CSA represents a unique mechanism in terms of catalytic residues, reaction catalysed and overall fold. Each entry can then be understood as a homologous family of thousands of enzyme sequences that follow the same mechanism.

In this paper, we describe a newly developed method to compare enzyme mechanisms that is based on the similarity of the bond changes and electronic transfers of each catalytic step. We discuss how different parameters of the method can be tweaked and how those affect the results. To demonstrate some practical applications of this similarity measure, we conducted a pairwise comparison of all the mechanisms annotated in the M‐CSA.

Among other results, we show how we can measure mechanistic chemical diversity across enzyme families, and a way to identify both common and unique types of catalytic events. By allowing us to cluster enzymes in accordance with their mechanism similarity, independently of their overall sequence, fold, and reaction, the method is useful for studying the relationships between these data. For example, it can be used to identify convergent evolution in enzymes that follow the same mechanism but have different folds, or divergent evolution in related enzymes that catalyse different overall reactions following a similar mechanism. Other applications, as well as current limitations and avenues for improvement, are addressed in the conclusions.

## Results

By analysing enzyme function at the mechanistic level of detail, it is possible to tackle questions that go beyond what can be learned solely from protein structure or their reactions. Unlike reaction data (a description of just the overall reactants and products), mechanisms tell us about the intermediates and transition states of the chemical steps the enzyme employs, which cover a fundamental understanding of catalysis. The difference between reaction and mechanism can be appreciated by noting that some enzymes can catalyse different reactions using essentially the same mechanism and others are able to catalyse identical or similar reactions using completely different mechanisms. Similar considerations might be made about enzyme structure and its relationship with reaction and mechanism.

The method developed in this work enables, for the first time, the systematic consideration of these questions as applied to hundreds of mechanisms across many enzyme families: How diverse is the chemistry happening in the active site of enzymes? How similar are the mechanisms of characterised enzymes? How does the similarity of enzyme mechanisms correlate to enzyme reaction and structure? And finally, what do these correlations tell us about enzyme evolution? These questions guide the analysis presented in the following sections.

### Data representation for enzyme mechanisms

**Fig. 1:**
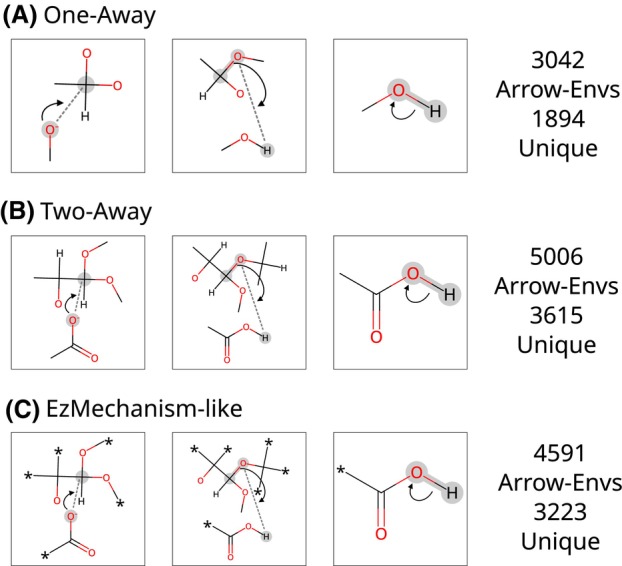
Representation of an enzymatic catalytic step using three different arrow‐environment definitions. Each group of horizontal arrow‐envs are a sequence of arrows that define the first step of the alpha‐amylase mechanism, as depicted in Fig. 2. (A) One‐away arrow‐envs include a single shell of atoms around the reaction centres. (B) Two‐away arrow‐envs include two shells of atoms around the reaction centres. (C) EzMechanism‐like arrow‐envs also include two shells of atoms around the reaction centres but C and H atoms in the second shell are considered equivalent. These centres are depicted in the figure as asterisks.

We started by creating a representation of enzyme mechanisms that is suitable to calculate their similarity. As the smallest unit of the comparison, we defined a new data entity called an ‘arrow‐environment’ (arrow‐env, see Fig. 1), which represents one curly arrow and the set of atoms and bonds involved in the electronic transfer that the arrow defines.

**Fig. 2:**
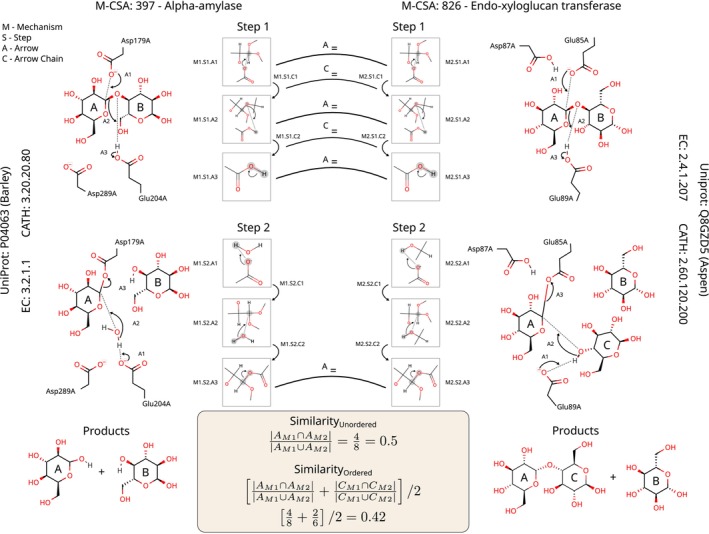
The enzyme mechanism similarity method developed in this work, detailed for two enzymes, shown side by side. The first enzyme, alpha‐amylase, catalyses the hydrolysis of an alpha‐glycosidic bond, while the second, endo‐xyloglucan transferase, breaks and then reforms the same type of bond to achieve the swapping of two glucose moieties (see depiction of the products at the bottom). Both mechanisms are composed of two catalytic steps, which in turn can be decomposed in three arrow‐envs. Identical arrow‐envs are linked with a line labelled ‘A_=_’ and identical sequences or chains of curly arrows are identified with a line labelled ‘C_=_’. Two variants of the similarity method, and how they are calculated, are shown in the box at the bottom.

To represent a catalytic step, all the arrow‐envs in that catalytic step are considered together, forming a graph where each arrow‐env is a node and arrows that follow each other are linked by a directed edge (see the sequences of arrow‐envs in Fig. 2 for an example). An arrow‐env is defined as ‘following another’ if its tail touches atoms that are being touched by the tip of the previous arrow. The ordering of arrow‐envs does not denote a temporal sequence since, by convention, all transformations in each catalytic step are concerted (otherwise they would define another catalytic step). Since chains of arrows in a catalytic step are not necessarily linear, they can form cycles or other types of structures; a graph representation is necessary. Mechanisms, in turn, can be represented as a linear sequence of the graphs that represent each catalytic step.

Arrow‐envs definitions are flexible in the number and type of atoms they include. For example, arrow‐envs shown in Fig. 1A contain only one shell of atoms around the reaction centres (the atoms directly involved in the electron transfer, highlighted in grey). ‘Two‐away’ arrow‐envs, shown in Fig. 1B, include the reaction centres plus atoms up to two bonds away. Figure 1C shows a more complex definition where second‐shell carbon and hydrogen atoms are specified as equivalent, since these two types of atoms should not have different contributions to the reaction at this distance. The EzMechanism‐like definition is inspired by the ‘rules of catalysis’ derived for the EzMechanism software. Other arrow‐env definitions might be created by adding more layers around the reaction sites or by considering other equivalent atom types. As discussed below, more specific definitions will produce fewer matches against other mechanisms and yield overall lower similarity scores.

The number of arrow‐envs generated for a given set of mechanisms can be a measure of the chemical diversity within that dataset, i.e., the larger the number of arrow‐envs, the larger the chemical diversity. Looking at specific arrow‐envs is also informative. If the same arrow‐env is observed in many enzyme mechanisms, it describes a type of chemistry that is common in enzyme catalysis. Conversely, arrow‐envs that are only seen once represent rare chemistry that is not observed elsewhere in the dataset. Note that, in the context of this study, ‘rare chemistry’ means that a certain type of transformation is not seen in other unrelated enzyme families, but it can still be quite common if that family has many members using the same mechanism. It is assumed that homologous enzymes that have the same catalytic residues and catalyse the same reaction also follow the same mechanism, and hence, to avoid redundancy, these are grouped together in one M‐CSA entry. Table 1 shows the overall number of mechanisms, steps and arrows in the M‐CSA mechanisms, as well as the different types of arrow‐envs that were extracted from those.

**Table 1:** Observed number of mechanisms, catalytic steps and curly arrows in the current version of the M‐CSA database, used as the analysed dataset in this paper. The bottom section shows the number of generated arrow‐envs according to each definition and the number of arrow‐envs that are very common and rare. A table with this information detailed for each EC number is available in the Supporting Information.

	Count
Mechanisms	734
Catalytic steps	3036
Arrows	19 311

Arrow‐env type	All	Common (seen in more than 20 steps)	Rare (only seen once)
One‐away	3042	94	1894
Two‐away	5006	47	3615
EzMechanism‐like	4591	52	3223

### Chemical diversity in enzyme mechanisms

The active sites of enzymes are composed of a small number of amino acids and cofactors, so redundancy in their catalysed chemistry is expected, and this is confirmed by our results. About 3000 arrow‐envs are sufficient to cover 19 311 actual curly arrows in 3036 catalytic steps and 734 mechanisms. This is the total number of One‐away arrow‐envs (Fig. 1A), which include a single layer of atoms around the reaction centres and are the less specific of the three definitions. One‐away arrow‐envs will yield more matches when comparing mechanisms, compared to the other definitions. On average, each one‐away arrow‐env matches more than six curly arrows, i.e., the chemical event described by the arrow‐env is observed more than six times across the database. Two‐away arrow‐envs lead to more stringent matches, and a larger number of them can be created (5006) to cover for the same M‐CSA dataset. On average, each two‐away arrow‐env matches approximately 3.9 curly arrows, or catalytic steps. Finally, matches of EzMechanism‐like arrow‐envs, where C and H atoms that are two bonds away are deemed equivalent, are slightly more generic than the two‐away definition. For this reason, there are fewer of them, 4591, and they match, on average, a slightly larger number of curly arrows, 4.2.

The distribution of arrow‐envs across catalytic steps and mechanisms is not uniform. Some arrow‐envs are observed in dozens of mechanisms while others are exclusive to a single enzyme. For example, there are 94 one‐away arrow‐envs that are seen in more than 20 catalytic steps. These types of arrow‐envs correspond to common types of chemical transformations, such as protonation and deprotonation reactions involving water molecules and the acidic and basic amino acids. The most common arrow‐envs of each type are shown in Figs S1–S3 and can also be identified among all arrow‐envs from the M‐CSA database at www.ebi.ac.uk/thornton‐srv/m‐csa/arrow‐environments/.

Most surprisingly is the number of arrow‐envs that are specific to a single M‐CSA enzyme family, 1894 for the one‐away definition (62%), and 3615 (72%) and 3223 (70%) for two‐away and EzMechanism‐like, respectively. This result indicates that although many enzyme families (each M‐CSA entry is considered a representative of one enzyme family) share some chemical steps, they also catalyse chemistry unique to that family. This result matches our previous observation in the EzMechanism study [21] where we verified that about half of the enzyme families studied performed at least one unique chemical transformation.

### Mechanism similarity method

Figure 2 describes the mechanism similarity method developed in this work. As an example, the accepted mechanisms of alpha‐amylase and endo‐xyloglucan transferase are shown side by side. Both mechanisms have two catalytic steps that can be represented as graphs, where nodes represent individual curly arrows and their chemical environment (arrow‐envs), and the edges define the sequence of curly arrows. This graph representation of the steps, using two‐away arrow‐envs, is shown beside every step.

The starting point of the similarity method is the direct comparison of arrow‐envs. These are deemed equivalent if they have the same exact atoms (or equivalent atoms as defined by the SMARTS substring), bonds, and electron transfer information, and are considered different otherwise. Chirality is not considered; thus, catalytic steps that only differ in their stereochemistry are considered equal. In Fig. 2, equivalent arrow‐envs are indicated by a line with a ‘A=’ label.

To calculate the similarity between any two catalytic steps, we have implemented two simple algorithms that take advantage of the graph representation of the mechanism, which might include disjointed subgraphs or circular paths.

In the first algorithm, only the nodes of the graph (each representing an arrow‐env) are considered, but not their relative order. Step similarity is calculated as the Jaccard/Tanimoto index between the two sets of arrow‐envs, where a score of one indicates that all arrows are the same, and a score of 0 indicates that there are no shared arrow‐envs in this step. Mechanism similarity can be calculated in the same way by using the sets of the arrow‐envs of all catalytic steps. For the two mechanisms in Fig. 2, for example, there are four matching and four non‐matching arrow‐envs, which produce an ‘unordered’ similarity score of 50%.

In the second algorithm, the relative order of arrow‐envs is also considered by considering the edges of the graph representation described above. In Fig. 2, these edges are named arrow chains, and similar arrow chains are represented by a line labelled as ‘C=’. The score for this ‘ordered’ measure of similarity is obtained by taking the simple average between the Jaccard index of the sets of arrow‐envs and the Jaccard index of the sets of the chains of arrow‐envs (the edges in the graph). For the mechanisms in Fig. 2, for example, which share two out of six different arrow‐env chains and half of the arrow‐envs, this approach yields an overall score of 42%.

Depending on the two types of similarity methods and the three types of arrow‐envs explained in the previous section, there are six possible combinations of similarity scores. Each score is named after the type of arrow‐env followed by the similarity measure. ‘Two‐away ordered’, for example, considers atoms up to two bonds away from the reaction centres and uses the ordered similarity measure.

### Mechanism similarity across the M‐CSA

**Fig. 3:**
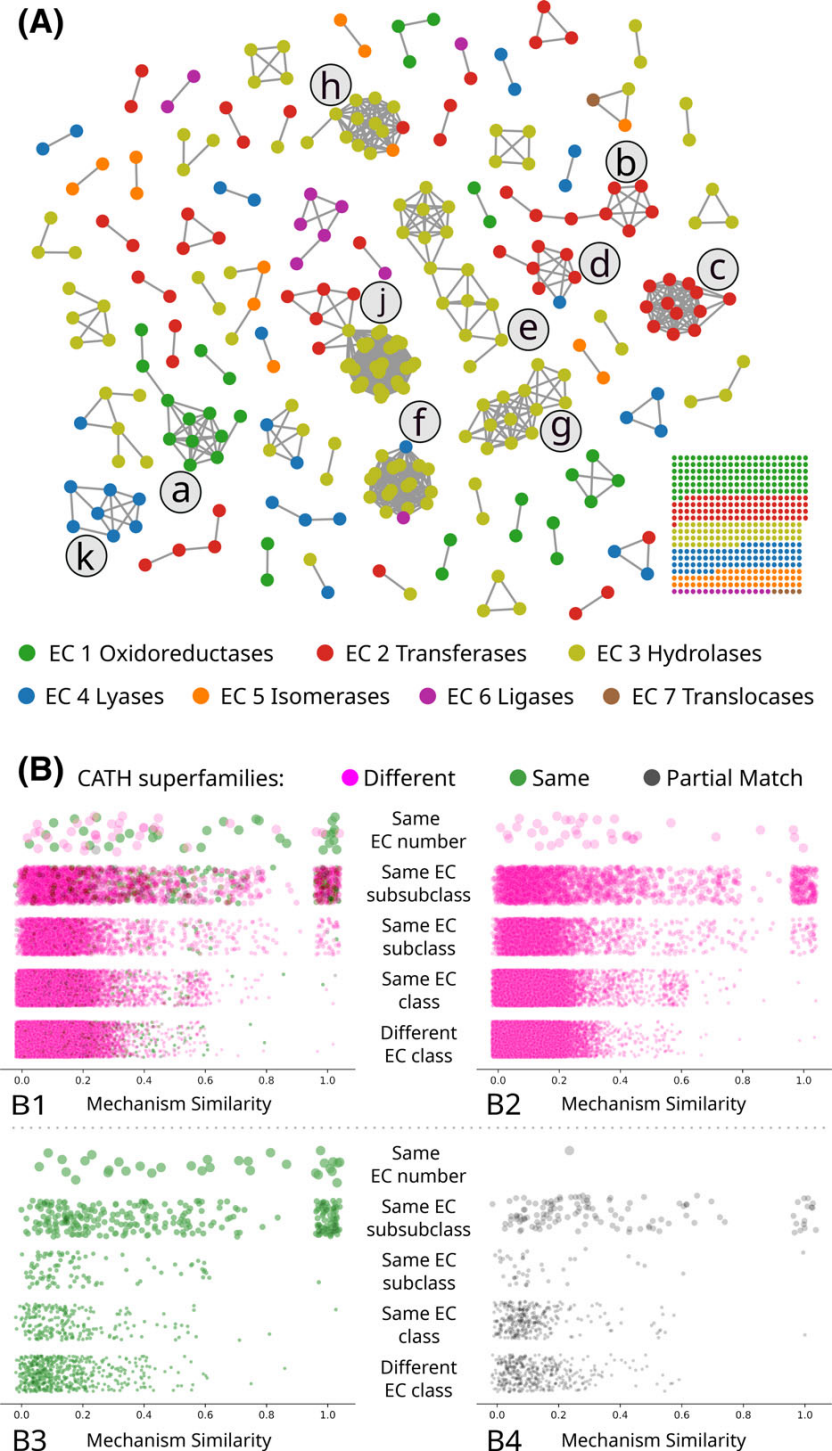
Pairwise comparison of enzyme mechanisms across the M‐CSA. (A) Clustering of similar enzyme mechanisms that are at least 50% similar according to the ‘One away – unordered’ score. Each node represents an enzyme mechanism, coloured according to their EC class. Nodes connected with edges are at least 50% similar. The dot matrix represents all the mechanisms in the database that do not share at least 50% similarity with another mechanism. (B) Mechanism similarity according to reaction similarity and evolutionary relationship. Each point is a comparison between two mechanisms. Nodes are coloured green (B3) if the two enzymes share the same CATH superfamilies, magenta (B2) if they do not share any CATH superfamily and grey (B4) if the enzymes share at least one but not all CATH superfamilies. Only domains with at least one catalytic residue are considered. Jittering was applied on all plots to increase the visibility of the dots. Panel B1 is constituted by the superposition of all the points in B2, B3 and B4.

Figure 3A shows the result of a pairwise similarity study that includes the 734 mechanisms in M‐CSA. Mechanisms are indicated as spheres, coloured by EC class, and edges are drawn between mechanisms that are at least 50% similar according to the ‘One‐away unordered’ score. The spheres on the bottom right represent mechanisms that are unlike any other mechanisms in the database, using this threshold and score. We stress again that, by design, the M‐CSA does not include homologous enzymes that follow the same mechanism to catalyse the same reaction, using the same active site. These are the majority of ‘similar’ enzymes in nature and would completely dominate an analysis like this but would also make it quite trivial. In the M‐CSA, we focus instead on examples of enzymes that have at least one difference in one of those variables, which is where the interesting cases of enzyme evolution occur.

The figure shows that clusters of highly similar mechanisms tend to belong to enzymes that catalyse the same type of chemical reaction (with some notable exceptions) according to their EC class. Some of the bigger clusters, labelled in the figure with lowercase letters, include: (a) several alcohol dehydrogenases (EC:1.1.1.‐), which are evolutionarily unrelated but all use NAD to perform redox reactions on different substrates; (b) many hexosyltransferases (EC: 2.4.1.‐) and two pentosyltransferases (EC: 2.4.2.‐); (c) several, mostly unrelated, phosphotransferases of alcohol groups (EC:2.7.1‐) and serine/threonine kinases (EC: 2.7.11.‐); (d) five Nucleotidyltransferases (2.7.7.‐) together with one diphosphotransferase (EC: 2.7.6.3) and adenylate cyclase (EC: 4.6.1.1) with different domain architectures but some shared domains; (e) several peptidases (EC:3.4.‐.‐) and other hydrolases acting on other carbon‐nitrogen bonds (EC:3.5.‐.‐) or on ester bonds (EC:3.1.‐.‐) all using a Ser residue as a nucleophile and mostly unrelated; (f) a cluster with several cysteine peptidases (also EC:3.4.‐.‐) that also includes a GMP synthase (EC: 6.3.5.2) and anthralinate synthase (EC: 4.1.3.27); (g) a series of unrelated peptidases (EC:3.4.‐.‐) and one carbon‐nitrogen hydrolase (EC:3.5.‐.‐) that use hydroxide as the main nucleophile, most commonly stabilised with metal ions; (h) many glycosylases (EC:3.2.‐.‐) together with an isomaltulose synthase (EC:5.4.99.11) and a xyloglucan:xyloglucosyl transferase (EC:2.60.120.200); (k) Several dehydratases (EC:4.2.1.‐) of open chain mono‐saccharides and other similar molecules.

Overall, these results show that enzymes that have similar mechanisms, tend to catalyse similar reactions. This is obviously the case in divergent evolution, where small changes in the protein sequence typically lead to similarly minor changes in mechanism and the reaction, but it is also observed in pairs of convergent enzymes, as is the case of alcohol dehydrogenases. Nevertheless, the inverse is not necessarily true, i.e. unrelated enzymes that catalyse the same reaction can do so using different mechanisms. Our method can easily distinguish between these different types of convergent evolution. A well‐known example of convergent evolution using different mechanisms includes the different types of proteases: cluster e—serine proteases; cluster f—cysteine proteases; and cluster g—metaloproteases.

In the ‘Mechanism Comparison’ page of the M‐CSA website (www.ebi.ac.uk/thornton‐srv/m‐csa/mechanism‐comparison/) it is possible to generate the same type of representation using different filters and for the other scores. Edges can also be coloured by EC or CATH similarity. Most of the protein pairs in the clusters discussed above are formed by domains belonging to different CATH superfamilies (M‐CSA does not store enzymes with the same mechanism, active site, and fold to avoid redundancy). Upon clicking on a node, this page will also show the mechanism for that enzyme and if an edge is clicked, both mechanisms will be shown in a manner that allows for a comparison to be made, similarly to what is shown in Fig. 2.

### Mechanism, reaction and structure similarity

Figure 3B shows the mechanism similarity for pairs of enzyme families according to differences in chemistry (using the EC classification) and overall structure (using the CATH classification). Each circle represents a pair of enzymes, which is coloured in green if their respective domains share the same superfamilies (top left bottom left), in grey if they share some but not all superfamilies (top left and bottom right), and in magenta if there is no overlap (top left and right). A shared structural superfamily (fourth level of the CATH classification) is indicative of a common evolutionary ancestor. Table 2 complements these plots and includes data for the two‐away and EzMechanism‐like definitions. The 734 mechanistic entries in M‐CSA map to 845 catalytic CATH superfamilies, including 491 catalytic CATH domains (each with at least one catalytic residue).

**Table 2:** Breakdown of the pairwise mechanism similarity across all mechanisms in M‐CSA, for the three arrow‐env definitions, using the unordered scoring method. CATH superfamily similarity should be understood as overall fold similarity, and EC subsubclass similarity is used to represent the similarity of the chemical reactions.

Mechanism similarity cut‐off	> 80%	> 50%	> 30%	> 15%
One‐away				
Mechanisms with matches (out of 734)	135 (18.4%)	284 (38.7%)	452 (61.6%)	656 (89.4%)
Matching pairs of mechanisms^a^	360	962	2112	12 284
Same CATH, same EC subsubclass^b^	112	184	253	347
Same CATH, different EC subsubclass	6	61	186	546
Different CATH, same EC subsubclass	178	329	552	1006
Different CATH, different EC subsubclass	64	388	1121	10 385
Two‐away				
Mechanisms with matches (out of 734)	89 (12.1%)	165 (22.5%)	302 (41.1%)	566 (77.1%)
Matching pairs of mechanisms^a^	190	319	667	4623
Same CATH, same EC subsubclass^b^	54	89	142	265
Same CATH, different EC subsubclass	1	2	44	228
Different CATH, same EC subsubclass	102	141	277	668
Different CATH, different EC subsubclass	33	87	204	3462
EzMechanism‐like				
Mechanisms with matches (out of 734)	95 (12.9%)	205 (27.9%)	339 (46.2%)	590 (80.4%)
Matching pairs of mechanisms^a^	221	484	863	5564
Same CATH, same EC subsubclass^b^	66	123	167	288
Same CATH, different EC subsubclass	1	22	87	314
Different CATH, same EC subsubclass	119	220	346	746
Different CATH, different EC subsubclass	35	119	263	4216

^a^ Total of 269 011 pairwise comparisons.

^b^ The M‐CSA dataset is non‐redundant and does not include enzymes with the same mechanism, active site and fold. In nature, where homologues usually have identical mechanisms, these would be the majority of matches if included.

The number of unrelated enzymes in M‐CSA (magenta circles) that catalyse the exact same reaction (same EC number) is low, but there are many examples of enzymes belonging to the same EC subsubclass (EC 3rd number). Typically, a subsubclass aggregates enzymes with the same type of chemical transformation but applied to different substrates. The distribution of mechanism similarity for this group is bimodal with peaks at the extremes, where the left side is populated by enzymes that converged to catalyse the same type of reactions using a different mechanism, and pairs on the right converged to use a similar mechanism (‘Different CATH, same EC subsubclass’ in Table 2).

As reaction similarity decreases, to the same EC subclass (EC 2nd number), the same EC class (EC 1st number), and finally different EC class, the distribution of similarity scores becomes increasingly concentrated on the left side (different mechanism). Nevertheless, for all these groups, there is still a sizeable number of pairs of mechanisms with considerable similarity (even a score of 30%, might correspond to 1 or 2 equal catalytic steps, for example). The thick distribution between 0% and 25%, even for mechanisms of enzymes of different EC classes, indicates that at least some mechanism similarity can commonly be expected, due to the arrow‐envs describing common chemical events, as explained above.

Table 2 presents another way to look at the results. At high levels of mechanism similarity (80%), most pairs of matching enzymes are either the result of convergent evolution (different CATH, same EC subsubclass) or divergent evolution to catalyse slightly different reactions (same CATH, same EC subsubclass). It is important to note that, by design, and to avoid redundancy, M‐CSA does not include identical mechanisms for related enzymes that also have an identical active site. For a dataset such as Swiss‐Prot or UniProt, it would be expected that most pairs of related enzymes with identical EC numbers or subsubclass would have a mechanism similarity score near 100%. Nevertheless, both the table and the plot (green circles in Fig. 3B) show that divergent evolution happens at the mechanistic level and that reaction dissimilarity increases are accompanied by more differences in the mechanism.

As the threshold of mechanism similarity decreases (to 30% and 15%), the number of random matches increases, and the number of unrelated enzymes catalysing unrelated reactions dominates the number of total matches.

## Discussion

We developed a new methodology to calculate enzyme mechanism similarity based on the curly‐arrow diagrams of their catalytic steps. The information on these diagrams is transformed into a graph representation of the catalytic steps, where nodes represent individual curly arrows and their chemical environment (arrow‐envs), and the edges define the sequence of curly arrows. Each arrow‐env contains information about the atoms and bonds of the reaction centres and surrounding atoms, including their atomic charge and bond types (from which the atomic hybridizations can be inferred).

Variants of the similarity method were constructed by varying the size and type of the chemical environment around the curly arrows. Smaller arrow‐envs are less specific and lead to more matches. Larger arrow‐envs are more specific, and more of them are necessary to cover a given dataset, such as the M‐CSA database. They produce fewer but more meaningful matches by including all the atoms and chemical groups relevant for the chemical reaction. An ideal arrow‐env definition would minimise the number of arrow‐envs while producing only meaningful matches from a chemical point of view. The EzMechanism‐like definition is a step in that direction, since the existence of either a C or H atom two bonds away from an electronic transfer probably has a small effect on the energetics of the reaction. Other definitions can be formulated containing additional equivalent atoms or groups, such as certain metallic ions or atoms belonging to the same periodic table group. Ideally, this choice should be guided by energetic considerations, as derived from computational chemistry methods or experimental data.

We used the newly developed method to perform a broad analysis of the mechanisms stored in the M‐CSA database in terms of their mechanism, reaction, and structure similarity. The method proved useful to group enzymes that, as expected, have the same reaction and mechanism, and was also able to automatically separate groups of enzymes that catalyse the same type of reaction using a different mechanism (such as Ser, Cys, and metal proteases). Other cases were identified where a similar mechanism is associated with both different reactions and folds. Overall, this simple analysis unearthed the complexity of the mechanism space in terms of enzyme function and evolution and shows the need for deeper studies on these relationships across families and clusters of similar mechanisms.

The current method presents a novel way of addressing a complex problem and as such has highlighted possible avenues for future development. Firstly, current scores do not consider any information related to non‐covalent interactions, which are, in most enzymes, responsible for stabilising the transition states. This information is available in M‐CSA but would require the inclusion of the 3D location and orientation of these residues in the algorithm. Another limitation is related to the non‐canonical representation of the mechanisms, since in some cases, the same electronic transfer in the same active site might be drawn differently (such as the electron flow moving through an aromatic ring, which can be drawn on one side or the other). The current method only considers one possibility at a time (the one drawn in the curly‐arrow diagram), which might lead to lower similarity scores. A third place for improvement, as already discussed, will be the creation of arrow‐env definitions that are more chemical aware, or specific to distinct types of analyses. Finally, while our implementation of the similarity method has only been applied to mechanisms already present in the M‐CSA, we plan to develop a new search tool where a user defined mechanism can be searched and compared against the mechanisms in M‐CSA, or potentially to other datasets.

We expect this and other upcoming mechanism similarity methods to become indispensable in the near future, as the number of studies of enzyme mechanisms keeps increasing. Adding to the traditional biochemical, structural, and computational approaches, this increase will be fuelled by new advances such as time‐resolved x‐ray crystallography [22] and new simulation approaches [23] coupled with the great number of available structures modelled using machine learning methods [24, 25, 26]. Certain questions that should be answered for every new mechanism studied will become increasingly difficult to answer; otherwise: How novel is this mechanism? Is this or a similar mechanism found in related or unrelated enzymes? How different is this mechanism proposal from other proposals for the same enzyme?

Mechanism similarity measures will also be essential for the development of automated methods to study enzyme mechanisms through computational means. We believe such automated methods will be the only way to characterise, on a large scale, the complex mapping between the protein sequence space and the enzyme reaction space. Knowing how past and future mutations impact reaction mechanisms will be key to understanding enzyme evolution and the design of new biocatalysts.

## Materials and methods

### Arrow‐environment representation

Each arrow environment is saved as a SMARTS [27] expression containing the atoms and bonds used in its definition, including information about their atomic charges and bond types. The position of the curly arrow is added to the SMARTS string as a two‐digit atom map according to the following rules: (a) the first digit of the atom map, either 1 or 2, refers to the number of moving electrons; (b) the second digit indicates whether the atom is touched by the tail of the arrow (1), its tip (2) or both (3). If the arrow tail or tip is interacting with a bond, then both atoms of that bond are considered. For example, the SMARTS expression for the arrow‐env on the upper left corner of Fig. 1 is ‘[#6]‐[#8&‐:23].[#8]‐[#6:22](−[#8])(−[#1])‐[#6]’. The SMARTS and pictorial representations of every arrow‐env extracted from the mechanisms currently available in the M‐CSA database are available at www.ebi.ac.uk/thornton‐srv/m‐csa/arrow‐environments/.

### Data and programming details

The data used during the development of the method and in the pairwise comparison comprises a set of detailed annotations of 734 enzyme mechanisms, as currently available in the M‐CSA database. Each mechanism stored in M‐CSA might be followed by thousands or millions of enzyme homologues. We avoid this redundancy by not including in the database enzyme mechanisms that follow the same catalytic steps and catalytic residues as performed by the same overall fold. At present, these mechanisms cover about 64% of all EC subsubclasses, with the following distribution across EC classes: EC1–75 of 148 subsubclasses covered, 51%; EC2–30 of 39, 77%; EC3–48 of 66, 73%; EC4–17 of 17, 100%; EC5–18 of 19, 95%; EC6–10 of 13, 77%; EC7–3 of 10, 30%. See Table S1 and [28] for a more detailed analysis of the M‐CSA coverage of EC space.

Of particular importance for this study are the two‐dimensional curly‐arrow diagrams of the catalytic steps of each mechanism, which show the rearrangement of electrons in the active site, from which the formation and cleavage of bonds can be inferred (see the four catalytic steps in Fig. 2). These diagrams are stored in the database as marvin document files (.mrv), a custom xml schema used by the marvin software [29], and are publicly available through the M‐CSA API.

Scripts to parse the mechanism files, to implement the comparison method and to perform the analysis were developed in Python. The Django web framework was used to communicate with the M‐CSA PostgreSQL database and to create the back end of the new arrow‐environment pages at www.ebi.ac.uk/thornton‐srv/m‐csa/arrow‐environments/ and the mechanism comparison page, available at www.ebi.ac.uk/thornton‐srv/m‐csa/mechanism‐comparison/. The latter uses cytoscape.js [30] to show a pairwise comparison graph (see Fig. 3A) and custom JavaScript code to control the available options and to filter the mechanisms and type of score being shown.

## Conflict of interest

The authors declare no conflict of interest.

## Author contributions

AJMR, IGR, NB and JMT conceived and designed the study. AJMR developed the new software and performed the analysis. IGR, NB, PAF, MJR and JMT contributed to discussions and provided guidance throughout the project. PAF, MJR and JMT provided the computational resources. AJMR wrote the first draft of the manuscript, and IGR, NB, PAF, MJR and JMT revised and edited the manuscript.

## Peer review

The peer review history for this article is available at https://www.webofscience.com/api/gateway/wos/peer‐review/10.1111/febs.70106.

## Supporting information


**Fig. S1.** The most common ‘one‐away’ arrow environments. The number below each arrow‐env is the number of catalytic steps where the arrow‐env is observed.
**Fig. S2.** The most common ‘two‐away’ arrow environments. The number below each arrow‐env is the number of catalytic steps where the arrow‐env is observed.
**Fig. S3.** The most common ‘EzMechanism‐like’ arrow environments. The number below each arrow‐env is the number of catalytic steps where the arrow‐env is observed.
**Table S1.** Observed number of mechanisms, catalytic steps and curly arrows in the current version of the M‐CSA database, used as the analysed dataset in this paper, decomposed for each EC class.

## Data Availability

The curly‐arrow diagrams for all catalytic steps are accessible through the M‐CSA API. All generated arrow environments can be found at www.ebi.ac.uk/thornton‐srv/m‐csa/arrow‐environments. Furthermore, mechanism comparison data is available via a graphical user interface at www.ebi.ac.uk/thornton‐srv/m‐csa/mechanism‐comparison. For any further information regarding the data or code generated for this work, please contact the corresponding author.
