# Supplementary material for: Measuring catalytic mechanism similarity – a new approach to study enzyme function and evolution
Source: FEBS J. 2025 Apr 22;292(16):4200–10. doi: 10.1111/febs.70106 (PMC12366284; doi:10.1111/febs.70106)
Supplement: Supplementary file 1 — Fig. S1. The most common ‘one‐away’ arrow environments. The number below each arrow‐env is the number of catalytic steps where the arrow‐env is observed. Fig. S2. The most common ‘two‐away’ arrow environments. The number below each arrow‐env is the number of catalytic steps where the arrow‐env is observed. Fig. S3. The most common ‘EzMechanism‐like’ arrow environments. The number below each arrow‐env is the number of catalytic steps where the arrow‐env is observed. Table S1. Observed number of mechanisms, catalytic steps and curly arrows in the current version of the M‐CSA database, used as the analysed dataset in this paper, decomposed for each EC class. [file FEBS-292-4200-s001.pdf]

Supporting Information for

Measuring catalytic mechanism similarity – a new approach to study enzyme function and evolution

Antonio J. M. Ribeiro[1,2*,] Ioannis G. Riziotis[2], Neera Borkakoti[2], Pedro A. Fernandes[1], Maria J. Ramos[1], and Janet M. Thornton[2]

Table SI-I Observed number of mechanisms, catalytic steps, and curly arrows in the current version of the M-CSA database, used as the analysed dataset in this paper, decomposed for each EC class.

	EC 1	EC 2	EC 3	EC 4	EC 5	EC 6	EC 7	Total
Total number of entries	216	204	300	139	83	42	10	994
Entries with mechanism description	164	148	211	110	69	26	6	734
Number of EC sub-sub-classes	148	39	66	17	19	13	10	312
Sub-sub-classes covered in M-CSA	75 (51%)	30 (77%)	48 (73%)	17 (100%)	18 (95%)	10 (77%)	3 (30%)	201 (64%)
Number of catalytic steps	876	544	734	512	248	103	19	3036
Number of curly arrows	6388	3234	4336	3161	1498	532	162	19311
Number of one-away arrow environments	1507	706	553	727	392	138	54	3042*
Number of two-away arrow environments	1986	1032	974	1088	550	181	58	5006*
Number of EzMechanism-like arrow environments	1913	954	846	1033	511	175	57	4591*

*The total number of arrow environments is smaller than the sum of the columns because many arrow environments are seen in many mechanisms

SI Figures Legends

SI-1 - The most common "one-away" arrow-environments. The number below each arrow-env is the number of catalytic steps where the arrow-env is observed.

SI-2 - The most common "two-away" arrow-environments. The number below each arrow-env is the number of catalytic steps where the arrow-env is observed.

SI-3 - The most common "EzMechanism-like" arrow-environments. The number below each arrow-env is the number of catalytic steps where the arrow-env is observed.

Note: Arrow-environments created with the different algorithms might be identical if they do not have any atoms after the first shell around the reaction centres.

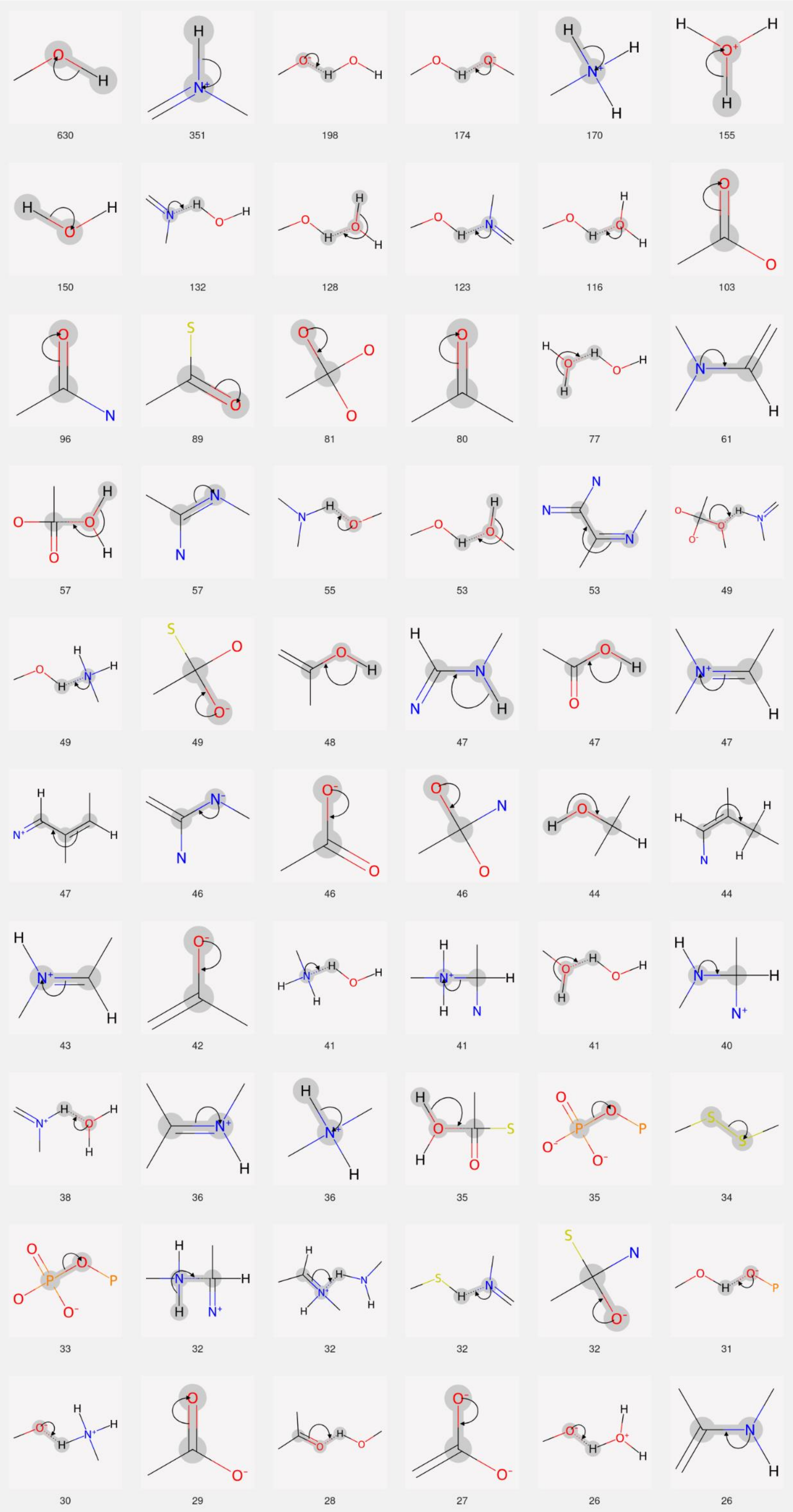
630 351 198 174 170 155
150 132 128 123 116 103
96 89 81 80 77 61
57 57 55 53 53 49
49 49 48 47 47 47
47 46 46 46 44 44
43 42 41 41 41 40
38 36 36 35 35 34
33 32 32 32 32 31
30 29 28 27 26 26

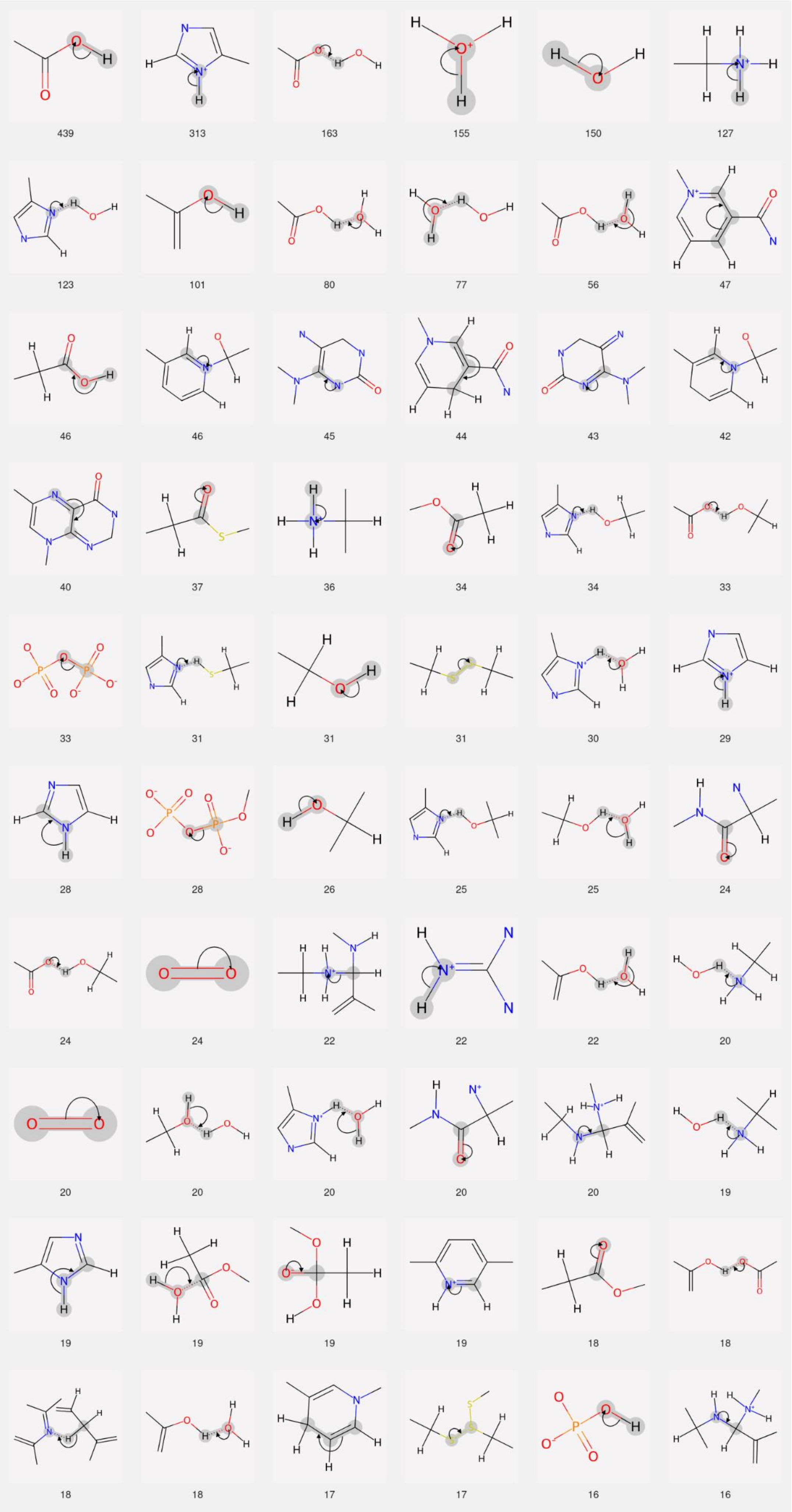

439 313 163 155 150 127
123 101 80 77 56 47
46 46 45 44 43 42
40 37 36 34 34 33
33 31 31 31 30 29
28 28 26 25 25 24
24 24 22 22 22 20
20 20 20 20 20 19
19 19 19 19 18 18
18 18 17 17 16 16

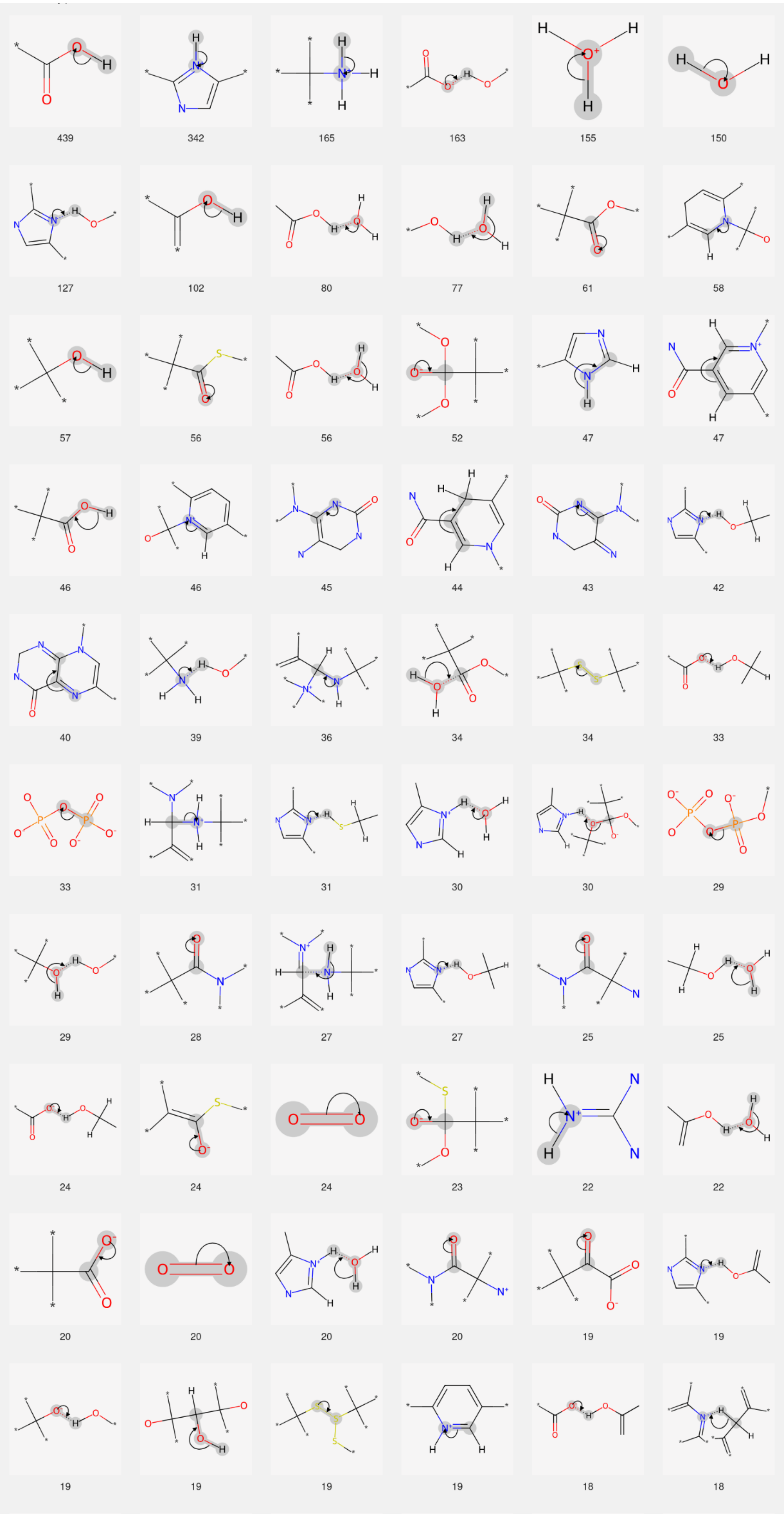
439 342 165 163 155 150
127 102 80 77 61 58
57 56 56 52 47 47
46 46 45 44 43 42
40 39 36 34 34 33
33 31 31 30 30 29
29 28 27 27 25 25
24 24 24 23 22 22
20 20 20 20 19 19
19 19 19 19 18 18
